# Single-cell sequencing analysis and multiple machine-learning models revealed the cellular crosstalk of dendritic cells and identified FABP5 and KLRB1 as novel biomarkers for psoriasis

**DOI:** 10.3389/fimmu.2024.1374763

**Published:** 2024-03-26

**Authors:** Zhiqiang Ma, Pingyu An, Siyu Hao, Zhangxin Huang, Anqi Yin, Yuzhen Li, Jiangtian Tian

**Affiliations:** ^1^ Department of Dermatology, The Second Affiliated Hospital of Harbin Medical University, Harbin, China; ^2^ Department of Ultrasound, Harbin Medical University Cancer Hospital, Harbin, China; ^3^ Basic Medical College, Harbin Medical University, Harbin, China; ^4^ Department of Ophthalmology, The Second Affiliated Hospital of Harbin Medical University, Harbin, China; ^5^ Department of Cardiology, The Second Affiliated Hospital of Harbin Medical University, Harbin, China; ^6^ The Key Laboratory of Myocardial Ischemia, Chinese Ministry of Education, Harbin, China

**Keywords:** single-cell sequencing analysis, machine learning, psoriasis, dendritic cell, biomarker

## Abstract

**Background:**

Psoriasis is an immune-mediated disorder influenced by environmental factors on a genetic basis. Despite advancements, challenges persist, including the diminishing efficacy of biologics and small-molecule targeted agents, alongside managing recurrence and psoriasis-related comorbidities. Unraveling the underlying pathogenesis and identifying valuable biomarkers remain pivotal for diagnosing and treating psoriasis.

**Methods:**

We employed a series of bioinformatics (including single-cell sequencing data analysis and machine learning techniques) and statistical methods to integrate and analyze multi-level data. We observed the cellular changes in psoriatic skin tissues, screened the key genes Fatty acid binding protein 5 (FABP5) and The killer cell lectin-like receptor B1 (KLRB1), evaluated the efficacy of six widely prescribed drugs on psoriasis treatment in modulating the dendritic cell-associated pathway, and assessed their overall efficacy. Finally, RT-qPCR, immunohistochemistry, and immunofluorescence assays were used to validate.

**Results:**

The regulatory influence of dendritic cells (DCs) on T cells through the CD70/CD27 signaling pathway may emerge as a significant facet of the inflammatory response in psoriasis. Notably, FABP5 and KLRB1 exhibited up-regulation and co-localization in psoriatic skin tissues and M5-induced HaCaT cells, serving as potential biomarkers influencing psoriasis development.

**Conclusion:**

Our study analyzed the impact of DC-T cell crosstalk in psoriasis, elucidated the characterization of two biomarkers, FABP5 and KLRB1, in psoriasis, and highlighted the promise and value of tofacitinib in psoriasis therapy targeting DCs.

## 1 Introduction

Psoriasis, a prototypical immune-mediated disorder (1), is characterized by erythematous skin lesions with scaling, which include vascular hyperplasia, destruction of vascular endothelial cells, and infiltration of inflammatory cell subpopulations, in addition to keratinocyte overgrowth, epidermal alterations and thickening (2, 3). While the pathogenesis of psoriasis remains incompletely understood, the overactivation of components within the innate and adaptive immune systems, particularly involving DCs and T cells, is currently deemed central to its etiology. Psoriasis involves three crucial inflammatory pathways: Th17 and Tc17 responses, Th1 and Tc1 responses, and the IL-36-neutrophil axis (1). With a genetic background and under the influence of external triggers, keratinocytes (KCs) are prompted to discharge endogenous DNA, RNA, and antimicrobial peptides (AMPs) such as LL-37 and human β-defensin-2/3. AMPs create complex structures with DNA or RNA, thus activating Toll-like receptors 7 and 8 (TLR7/8), thereby prompting plasmacytoid dendritic cells (pDCs) to initiate the production of IFN-α and IFN-β, subsequently stimulating myeloid dendritic cells (mDCs) to secrete pro-inflammatory factors. Natural killer T (NKT) cells and KCs also discharge various cytokines and chemokines, notably TNF-α, IFN-γ, IL-1, IL-6, and CXCL1, influencing mDCs (4). Changes in DCs have garnered increasing attention in the pathophysiology of psoriasis (5, 6). Activated DCs migrate to skin-draining lymph nodes, where their secretion of IL-23, TNF-α, IL-1β, and IL-12 promotes the differentiation of CD4+ naïve T cells into Th17, Th22, and Th1 cells, while IL-23 induces CD4-CD8- T cells to differentiate into Gamma-delta (γδ)T cells (7). Inflammatory cytokines secreted by these cells trigger signal transduction within KCs, resulting in gene transcription of cytokines and chemokines, thus initiating diverse immune pathways focused on IL-17 and IL-23 (1, 8). Previous studies revealed that T cells’ interaction with monocytes triggered specific DC subpopulations’ differentiation. The interplay of Th1 and Th17 cells with monocytes in skin lesions continued to promote DC formation (9, 10). DCs contribute to psoriasis pathogenesis and recurrence by eliciting T cell-associated inflammatory responses through the IL-23/IL-17 axis in response to inflammatory cytokines. Tissue-resident memory T (TRM) cells also contribute significantly to the pathogenesis and recurrence of psoriasis, as the sustained presence of IL-17-secreting CD8+ T cells and IL-22-secreting CD4+ T cells can result in chronic inflammatory plaque formation (11). Thus, it is hypothesized that the modulation of T cells by DCs is a pivotal factor in psoriasis pathogenesis.

Current challenges in psoriasis research encompass differential diagnosis, notably distinguishing it from inflammatory, infectious, and neoplastic diseases without relying on pathologic testing. For instance, plaque psoriasis is often misdiagnosed as candidiasis or fungal infections, while pruritic psoriasis can be mistaken for atopic dermatitis. Additionally, the early stages of cutaneous T-cell lymphomas closely resemble those of psoriasis, and identifying seborrheic dermatitis on the head and face poses challenges (1). Beyond diagnostic intricacies, research grapples with the diminishing efficacy of biologics and small molecule targeted agents, as well as the management of psoriasis co-morbidities. Therefore, it is important to investigate biomarkers in the progression of psoriasis.

In this study, we used bioinformatics tools such as single-cell sequencing data analysis and machine learning to identify the key molecular mechanisms associated with DCs in psoriasis, screened FABP5 and KLRB1 as new biomarkers in the diagnosis and treatment process, and investigated drugs with better efficacy in targeting the altered number of DCs, which provided new insights for the clinic. In conclusion, this study contributes to exploring the underlying pathogenesis of psoriasis and provides new strategies for more precise diagnosis and treatment.

## 2 Materials and methods

### 2.1 Datasets downloaded

The single-cell RNA sequencing data from 13 psoriasis skin samples and 5 normal skin samples, retrieved from the GSE151177 dataset in the Gene Expression Omnibus (GEO) database, were obtained for this study. Furthermore, the gene expression data and comprehensive clinical information of normal individuals and patients with psoriasis were acquired from the GSE41664, GSE85034, GSE117468, and GSE69967 datasets. It is important to emphasize that all the data reanalyzed in the current study were publicly available in previous reports, as shown in **Supplementary Table S1**.

### 2.2 Single−cell RNA−seq data preprocessing

Raw single-cell RNA sequencing (scRNA-seq) data underwent processing with the Seurat R package (Version 4.3.0.1) to eliminate low-quality cells and conduct data visualization. Unqualified cells (with fewer than 500 genes per cell and less than 5 cells per gene) were excluded, while mitochondrial genes and hemoglobin genes were omitted from subsequent analyses. In addition, the scRNA-seq data were normalized and scaled using Seurat after identifying the top 2000 highly variable genes (HGVs). The harmony R package (Version 0.1.1) was utilized to address sample batch effects. Principal component analysis (PCA) was subsequently performed, and 21 principal components (PCs) were selected for subsequent t-distributed stochastic neighbor embedding (tSNE) analysis. Cell types were annotated based on well-established marker genes, and Seurat was used to identify highly expressed genes within each cell cluster. Furthermore, the gene expression matrix and clinical features were extracted for further analysis.

### 2.3 Gene set functional analysis

The gene ontology (GO) enrichment analysis and Kyoto Encyclopedia of Genes and Genomes (KEGG) enrichment analysis of highly expressed genes in each cell cluster were conducted using the clusterProfiler R package (Version 3.17). Firstly, highly expressed genes or differentially expressed genes (DEGs) were derived from Seurat. Subsequently, these genes were enriched using the clusterProfiler package, employing a significance cutoff of p < 0.05. Moreover, gene set enrichment analysis (GSEA) was performed with the clusterProfiler (Version 3.17) and msigdbr (Version 7.5.1) R packages. The DEGs were sorted based on log2Fold Change and matched with gene names, followed by clusterProfiler analysis with default parameters. The enrichment results from multiple gene sets with p < 0.05 were visualized using the enrichplot R package (Version 3.17).

### 2.4 Cell-cell communication analysis

The gene expression matrix and clinical features of 20,076 cells were extracted using Seurat to investigate cell-cell communication. Specifically, we employed the CellChat R package (Version 1.1.0) to analyze annotated gene expression data based on the official workflow and default parameters. This analysis inferred receptor-ligand interactions by measuring the expression levels of ligands and receptors. The communication probability was assessed using the “computeCommunProb” function, and the signaling pathways with a significance level of p < 0.05 were visualized using the “netVisual_aggregate” and “netVisual_chord_gene” functions.

### 2.5 Cellular trajectory analysis

The cellular trajectory analysis was performed using the R packages CytoTRACE (Version 0.3.3) and monocle2 (Version 2.18.0). In summary, we constructed a KNN graph containing information on DCs and calculated a pseudo-temporal ordering using CytoTRACE. Subsequently, we visualized the graphs based on transcriptional diversity on a tSNE plot. Additionally, the gene expression matrix and clinical information were extracted using Seurat and imported to create an object in monocle2. For the subsequent trajectory analysis, genes with a mean expression ≥ 0.1 were selected. Differential expression genes (DEGs) with a q-value < 0.01 between the DCs clusters were subjected to dimension reduction using the “reduceDimension” function with default parameters. Finally, the cells were ordered and visualized using the “plot_cell_trajectory” function. Furthermore, the genes in each cluster underwent GO enrichment analysis using clusterProfilter, as previously described.

### 2.6 Identification of key markers in psoriasis cohort

Three machine learning algorithms, namely the least absolute shrinkage and selection operator (LASSO), random forest (RF), and support vector machines-recursive feature elimination (SVM-RFE), were employed to identify key genes involved in psoriasis development. In short, the psoriasis cohort was randomly divided into a training set and a test set, with a split ratio of 8:2, where the training set contains 109 samples and the test set contains 48 samples. Psoriasis and normal skin samples were analyzed using LASSO regression with 10-fold cross-validation, implemented in the R package glmnet (Version 4.1-7). A random forest model, based on marker genes of DCs, was constructed using the R package randomForest (Version 4.7-1.1). Furthermore, the SVM-RFE model, generated using the R package e1071 (Version 1.7-13), was used to rank the genes. The predictive value of these models was assessed using receiver operating characteristic (ROC) analysis, conducted with the R package pROC (Version 1.18.4).

### 2.7 Immune cell infiltration analysis

The analysis of immune cell infiltration was conducted using the R package xCell (Version 1.1.0). The xCell algorithm used the samples’ RNA-seq data to derive the immune cell index. Pearson’s correlation analysis was performed to assess the correlation coefficients between the expression of key genes and the infiltration levels of immune cells. All visualizations were generated using the R package ggplot2 (Version 3.4.2).

### 2.8 Pearson correlation analysis

The relationship between the expression of key genes and infiltrating immune cells was assessed using Pearson correlation analysis. Visualization of these relationships was performed using the R package ggcor (Version 0.9.8.1).

### 2.9 Cell culture and exposure to M5 stimulation

Human epidermal keratinocytes (HaCaT) were cultured in DMEM medium (Gibco) containing 10% fetal bovine serum (Hyclone, US) at 37°C in 5% CO2. *In vitro* modeling of psoriasis was achieved through the M5 induction method. HaCaT cells were seeded onto a 6-well plate until reaching 60–70% confluence, followed by a 24-hour serum-free DMEM starvation period. Subsequently, cells were stimulated for 48 hours with a medium containing IL-1α, IL-17A, IL-22, TNF-α, and oncostatin M (10 ng/mL for each cytokine, ProSpect, Israel).

### 2.10 mRNA isolation and quantitative reverse transcription PCR assays

The M5 HiPer Universal RNA Mini Kit (Mei5bio, China) was used to extract total RNA from tissues and HaCaT cells, and the Transcriptor First Strand cDNA Synthesis Kit (Roche) was used to create cDNA. The quantitative reverse transcription PCR (RT-qPCR) experiments utilized the NovoStart SYBR qPCR SuperMix Plus Kit (Novoprotein) on a Bio-Rad CFX96 Real-Time PCR Detection System. Standardized relative mRNA expression levels were determined using GAPDH values. **Supplementary Table S2** contains the primers used in this test.

### 2.11 Immunohistochemistry assays and semi-quantitative analysis

Three psoriasis lesions and three healthy skin tissues were fixed, dehydrated, and made into paraffin blocks, after which they were cut into slices of 5 μm thickness using a slicer (Leica Co., Ltd., HistoCore BIOCUT) and baked at 60°C for 20 min. After a 25-minute soak in xylene, the specimen underwent hydration with anhydrous alcohol, followed by sequential immersion in 95%, 80%, and 70% alcohol for 2 minutes each. It underwent three rinses with PBS for 3 minutes each. The specimen was then placed in a restorative solution at 95 degrees Celsius for 15-20 minutes to facilitate antigenic restoration, followed by natural cooling for 20 minutes. Following this, a serum blocking solution was applied, and the specimen underwent incubation at room temperature for 20 minutes. The application of FABP5 (D1A7T) Rabbit mAb (CST, #39926, 1:3600) and KLRB1/CD161 Monoclonal antibody (proteintech, 67537-1-Ig, 1:500) dropwise ensued, with overnight incubation at 4 degrees Celsius. The subsequent steps included color development, hematoxylin re-staining, routine dehydration, sealing, and microscopic scanning. For each group of immunohistochemistry result maps under 20x field of view, we selected 3 representative images for semi-quantitative analysis. The quantification of the stained area in each image and the integrated optical density (IOD) of the indicated markers were measured using Image J and assessed using the Average optical density (AOD = IOD/Area) method. Also, Image J was employed to assess the positive staining of KLRB1 and FABP5.

### 2.12 Fluorescence immunohistochemistry and quantitative image analysis

The paraffin blocks were sliced into thin slices of 5 μm thickness with a slicer and baked at 60°C for 1 hour. The portions were deparaffinized with xylene 3 times, each time for 10 min. Dehydrated with graded alcohol gradients (100%, 95%, and 70% stepwise for 5 min each time), washed twice with water, and microwaved, after which the sections were blocked with goat serum blocking solution (CWBIO, 01380/34021). After being washed with PBS and co-incubated with a secondary antibody for 10 min, the slices were blocked after treatment with Antifade Mounting Medium with DAPI (Beyotime, PO131), observed and photographed under a light microscope (CX23, OLYMPUS). Quantitative analysis was conducted using HALO’s (Indica Labs) Highpex FL (V4.2.3) and Area Quantification FL (V2.2) module to determine the percentage of positive cytoplasms and cells. The FABP5 to KLRB1 fluorescence ratio was quantified with Fiji (ImageJ), and the normalized ratios are graphically presented adjacent to the HALO analysis image.

### 2.13 Statistical analysis

All data processing was conducted using R software (Version 4.2.1) and Graphpad Prism (Version 9.5.1). The error bars in the figures represent the standard error of mean (SEM). The statistical analyses in the current study utilized the T-test and Wilcoxon test. A significance level of P < 0.05 was considered statistically significant.

## 3 Results

### 3.1 Single-cell expression atlas of psoriasis patients

**Figure 1:**
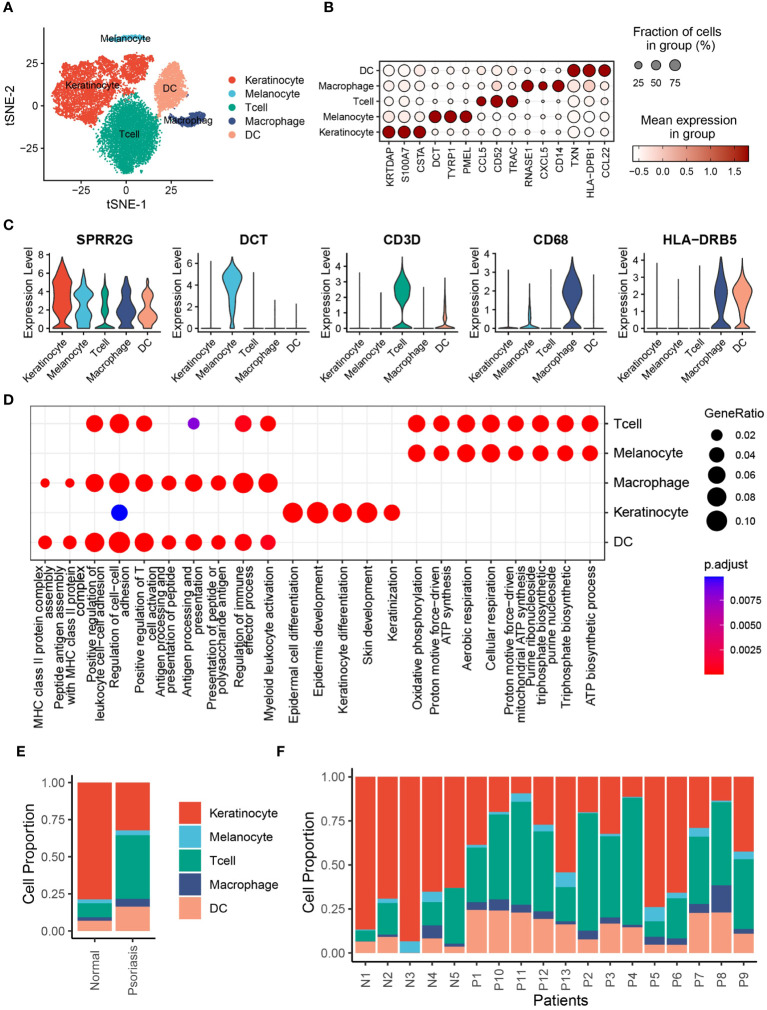
Single-cell expression atlas of psoriasis patients. **(A)** t-distributed stochastic neighbor embedding (tSNE) plots of 20076 cells extracted from healthy and psoriasis skin tissues. Cell types are labeled by colors. **(B)** Bubble plot depicting expression and percentage of positive cells in different clusters. **(C)** Violin plots depicting log normalised expression per cluster of key markers used in cluster annotation. **(D)** Dot plot showing the activated signalling pathways in different cell clusters. **(E)** Bar plot showing the proportion of different cell types in normal and psoriasis skin tissues. **(F)** Bar plot showing the proportion of different cell types in different samples.

To investigate the cell composition of psoriatic skin, we integrated and reanalyzed the scRNA-seq cohort obtained from GSE151177, including 13 psoriasis skin samples and 5 normal skin samples (**Supplementary Table S1**). After quality control, we excluded cells with low quality, including dead cells and doublets, and ultimately acquired 20076 cells retained for subsequent analysis (**Supplementary Figures S1A–F**). Then, we identified 2000 HVGs in 20076 cells (**Supplementary Figure S2A**), and integrated cells from different samples via “harmony” algorithm, which removed batch effects (**Supplementary Figure S2B**). Eventually, we observed 6 distinct psoriasis cell type clusters with K-means algorithm (**Supplementary Figure S2C**). Based on Pearson’s correlation analysis and universal marker genes, we annotated these 20439 cells as 5 distinct cell types, including KCs (8326 cells, marked with LCE3D, KRT5 and KRT14), melanocytes (633 cells, marked with DCT, TYRP1 and MLANA), T cells (7533 cells, marked with CD3D, CD3E and PTPRC), macrophages (959 cells, marked with CD68, CD163 and CD14), DCs (2988 cells, marked with LYZ and HLA-DRB5) (**Figure 1A**, **Supplementary S2D, E**). To further explore the additional marker genes of these cells, we performed differentially expression analysis and identified CSTA and KRTDAP as novel markers of KCs, whereas PMEL as novel markers of melanocytes (**Figures 1B, C**). To validate the biological functions of these cells, we performed gene set functional analysis. The results showed that the epidermal cell differentiation, KC differentiation and skin development pathways were activated in KCs, while MHC class II protein complex assembly and peptide antigen processing related pathways were activated in DCs and macrophages, which were consistent with existing literature (**Figure 1D**). We further performed cell composition analysis to further compare the cell composition between normal skin tissues and psoriasis skin tissues. The results revealed that KCs were significantly decreased, whereas DCs were significantly increased in psoriasis skin tissues, suggesting severe inflammation during psoriasis development (**Figures 1E, F**).

### 3.2 RNA splicing pathways were inactivated in KCs from psoriasis skins

To deepen our comprehension of the changes in KC function, we conducted further analysis on different subsets of KCs from normal and psoriasis skin tissues. Our findings revealed that KCs displayed heterogeneity and could be classified into 5 distinct subclusters (**Supplementary Figure S3A**). Notably, cluster 0 predominantly consisted of KCs from psoriasis skin tissues, while cluster 1 primarily contained KCs from normal skin tissues (**Supplementary Figures S3B–D**). We also identified marker genes specific to each subcluster of KCs. Cluster 0 was characterized by the expression of SPRR2D, SPRR2F, and SPRR2A, whereas cluster 1 exhibited high expression of KRT23, CCL20, and SPINK5 (**Supplementary Figure S3E**). Interestingly, the association of SPRR2A and SPRR2D with psoriasis has been previously confirmed (12), supporting the relevance of our findings. We further performed KEGG enrichment analysis to explore the functional implications of these keratinocyte subclusters. We observed a significant downregulation of RNA modification, RNA splicing, and spliceosome pathways in cluster 0 KCs compared to cluster 1 KCs. These findings align with recent studies highlighting the involvement of these pathways in psoriasis (13–15). To investigate the transcriptional changes between KCs from normal and psoriasis skin tissues, we identified differentially expressed genes (DEGs). Among these DEGs, DEFB4A was significantly upregulated in psoriasis (**Supplementary Figure S3F**). DEFB4A has been shown to mediate the activation of CCR6 Th17 cells, further supporting its role in psoriasis (16). Moreover, enrichment analysis revealed the activation of ATP metabolic process and purine nucleoside triphosphate metabolic process in psoriasis. These findings suggest that metabolic and immune disorders play important roles in the development of psoriasis (**Supplementary Figure S3G**).

### 3.3 DCs regulate T cells through the CD70/CD27 signaling pathway

**Figure 2:**
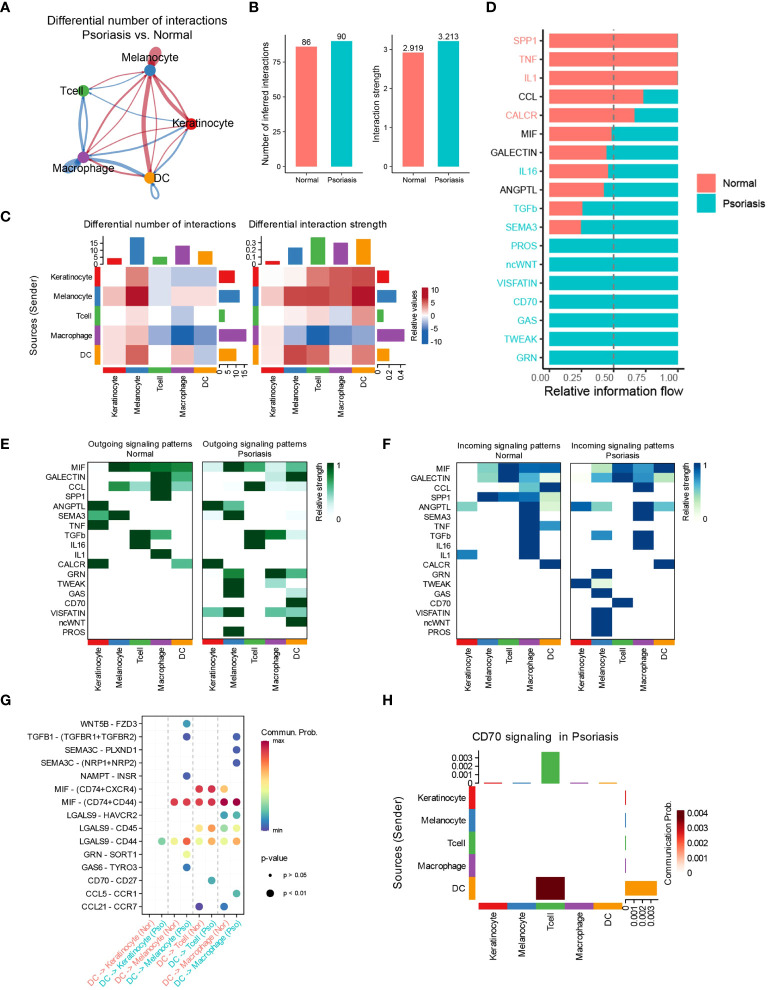
DCs regulate T cells through the CD70/CD27 signaling pathway. **(A)** Circle plot showing the differential number of interactions between normal tissues and psoriasis tissues. **(B)** Bar plots showing the number of inferred interactions and interaction strength in normal and psoriasis tissues. **(C)** Heat map showing the differential number of interactions and differential n interaction strength between normal tissues and psoriasis tissues. **(D)** Bar plot showing the relative information flow in normal and psoriasis tissues. **(E)** Heat map showing the outgoing signaling patterns in normal and psoriasis tissues. **(F)** Heat map showing the incoming signaling patterns in normal and psoriasis tissues. **(G)** Dot plot showing the potential mechanism of cell-cell communications from DCs to other cell types. **(H)** Heat map showing theCD70 signaling pathway in psoriasis tissues.

To investigate the potential mechanisms underlying the increased effects of DCs in promoting psoriasis, we employed the “CellChat” algorithm to analyze cell-cell communication among five distinct cell types. Our analysis revealed significant increases in the number of inferred interactions and interaction strength in psoriasis skin tissues (**Figures 2A, B**). Particularly, the interaction strength between DCs, T cells, and melanocytes was markedly enhanced, while the interaction strength between macrophages, T cells, and DCs was significantly inhibited (**Figure 2C**). Mechanistically, we observed a significant increase in the relative information flow of CD70, SEMA3, and TGF-β in psoriasis skin tissues. Conversely, the relative information flow of SPP1 and TNF was significantly decreased. These findings align with previous studies and support the involvement of these molecules in psoriasis (**Figure 2D**) (17–19). We further examined the outgoing and incoming signaling patterns between cells in normal and psoriasis tissues. The results revealed that the outgoing signals of CD70, GRN, ncWNT, and GAS from DCs were significantly increased in psoriasis. Additionally, the incoming signaling of CD70 in T cells was significantly increased. These findings suggest the importance of CD70-related pathways in the development of psoriasis (**Figures 2E, F**). Furthermore, through a more detailed analysis, we found that DCs regulate the functions of KCs, melanocytes, T cells, and macrophages via specific pathways. DCs utilize the LGALS9/CD44 pathways to influence KCs, the GAS6/TYRO3 pathways to interact with melanocytes, the CD70/CD27 pathways to modulate T cell behavior, and the CCL5/CCR1 pathways to regulate macrophages (**Figures 2G, H**).

### 3.4 DCs are heterogeneous in patients with psoriasis

**Figure 3:**
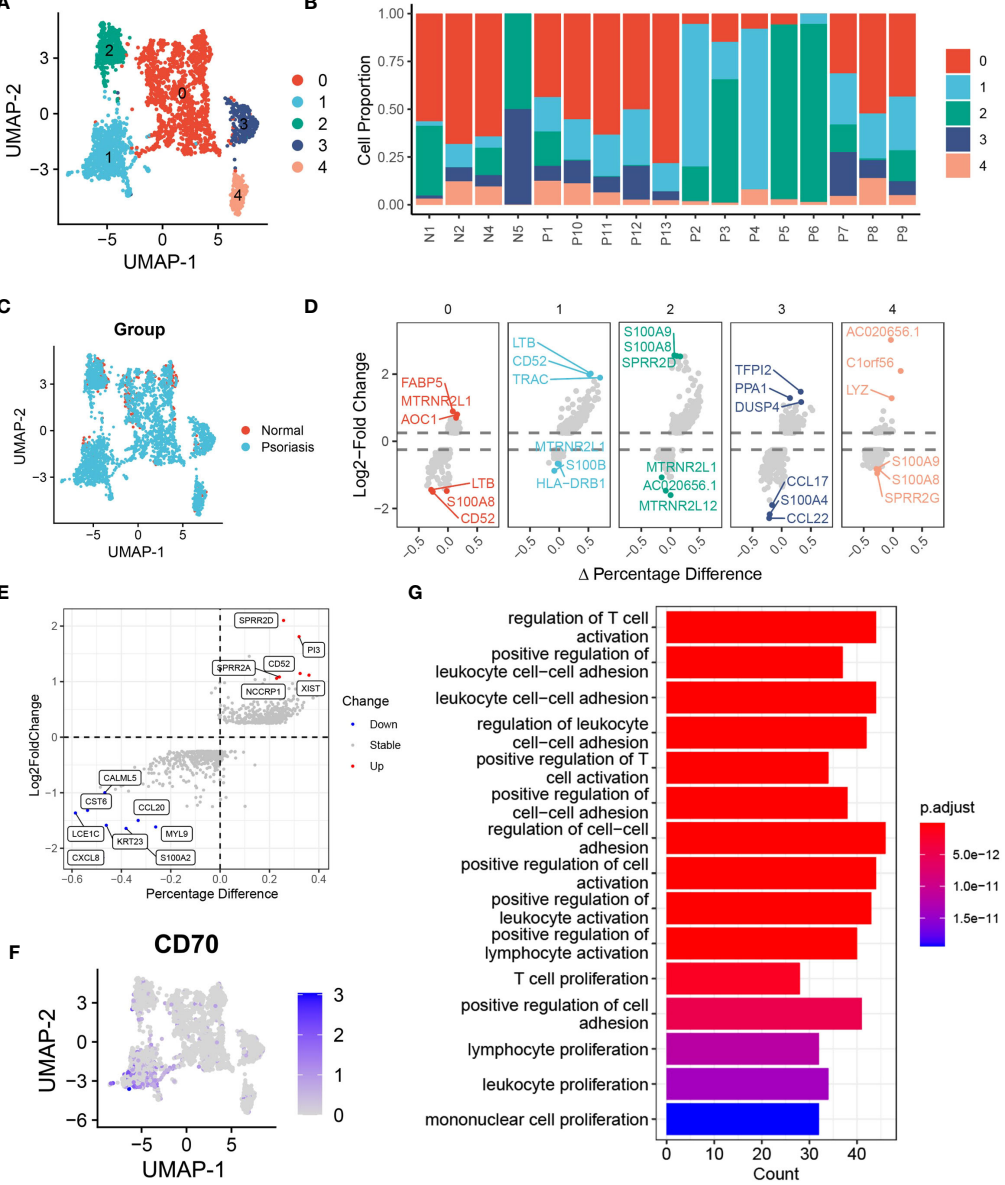
DCs are heterogeneous in patients with psoriasis. **(A)** tSNE plot showing the 2988 DCs in 5 distinct clusters with K-means algorithm. **(B)** Bar plot showing the DCs proportion in 4 normal skin tissues and 13 psoriasis skin tissues. **(C)** tSNE plot showing the 2988 DCs extracted from normal and psoriasis skin groups. **(D)** Volcano plot showing the highly expressed genes in 5 distinct DCs clusters. **(E)** Volcano plot showing DEGs of DCs between psoriasis and normal skin tissues. **(F)** Feature plot showing the expression of CD70 in DCs. **(G)** Bar plot showing the alternative pathways of DCs between psoriasis and normal skin tissues.

In our investigation of the functional heterogeneity of DCs in psoriasis development, we examined different subsets of DCs isolated from normal and psoriasis skin tissues. Our analysis using unsupervised clustering methods revealed 5 distinct clusters of DCs (**Figure 3A**, **Supplementary S4A**). Further analysis of cell proportions demonstrated that cluster 0 and cluster 2 DCs were primarily found in normal skin tissues, while cluster 1 DCs were primarily present in psoriasis skin tissues (**Figures 3B, C**, **Supplementary S4B**). To validate the differences observed among the clusters, we conducted a differential expression analysis and identified significant upregulation of LTB, CD52, and TRAC in cluster 1 DCs. These genes have been extensively associated with hyperinflammation (**Figure 3D**) (20–22). To gain further insights into the molecular mechanisms driving the observed differences, we performed differential expression analysis of DCs between psoriasis and normal tissues. This analysis revealed the overexpression of SPRR2D, CD52, and PI3 in DCs isolated from psoriasis skin tissues (**Figure 3E**). Notably, consistent with our previous findings, gene expression analysis confirmed the specific expression of CD70 in cluster 1 DCs, providing further support for the significant involvement of the CD70/CD27 signaling pathway in psoriasis development (**Figure 3F**). To better understand the functional implications of the DEGs and pathways in psoriasis-associated DCs, we conducted KEGG enrichment analysis. The results highlighted the activation of pathways involved in the regulation of T cell activation, positive regulation of cell adhesion, and T cell proliferation in DCs from psoriasis skin tissues (**Figure 3G**). These findings suggest that DCs promote psoriasis through the activation of T cells. Additionally, GSEA further supported our results, showing significant activation of adaptive immune response and lymphocyte activation in DCs from psoriasis skin tissues (**Supplementary Figure S4C**).

### 3.5 Pseudotime and single-cell trajectory analyses revealed the different cell fates of DCs

**Figure 4:**
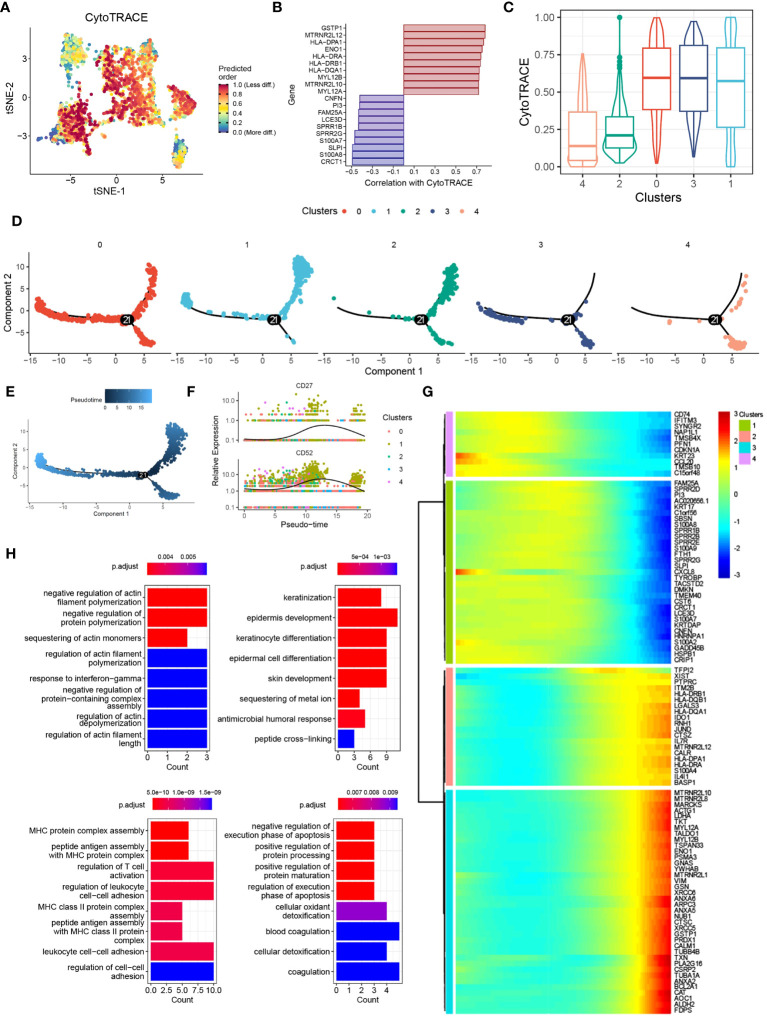
Pseudotime and single-cell trajectory analyses revealed the different cell fates of DCs. **(A)** tSNE plot showing the CytoTRACE score of DCs clusters. **(B)** Bar plot showing the genes correlated with CytoTRACE. **(C)** Box plot showing the CytoTRACE score of different DCs subclusters. **(D)** PCA plot showing the subclusters of DCs. **(E)** PCA plot showing the psedotime of different DCs subclusters. **(F)** Scatter plot showing the expression of CD27 and CD52. **(G)** Heat map showing the alternation of gene clusters. **(H)** Bar plot showing the biological functions of different gene clusters.

To investigate the functional transition of DCs during psoriasis development, we utilized the CytoTRACE algorithm and Monocle2 algorithm to assess their differentiation potential and evolutionary direction. The results highlighted the heterogeneous nature of differentiation potential and expression patterns among different DC clusters (**Figures 4A, B**). Specifically, cluster 1 DCs exhibited a higher CytoTRACE score, indicating greater cell stemness and proliferation rate (**Figure 4C**). Concurrently, the Monocle2 analysis revealed a significant increase in the expression of CD27 and CD52 during cell differentiation (**Figures 4D-F**). Notably, the CD27/CD70 signaling pathway has been extensively linked to the promotion of autoimmune diseases, whereas CD52 has been associated with inflammation suppression, suggesting that DCs may exert a dual mode of action on T cells (23, 24). To explore the potential molecular mechanisms underlying these observations, we conducted gene pattern analysis. The results demonstrated that gene clusters 1 and 2 were significantly downregulated, including genes such as CD74, CCL20, and PI3. Conversely, gene clusters 3 and 4 were markedly upregulated, incorporating genes such as XIST and FDPS (**Figure 4G**). These findings align with our previous results and suggest distinct transcriptional changes associated with the differentiation of DCs during psoriasis development. Functional enrichment analysis provided further insights into the roles of these gene clusters. Gene clusters 1 and 2 were associated with the regulation of actin filament polymerization and keratinization, which are processes relevant to skin homeostasis and barrier function. On the other hand, gene clusters 3 and 4 were linked to MHC protein complex assembly and the regulation of the execution phase of apoptosis (**Figure 4H**). These findings are consistent with previous knowledge and underline the functional alterations occurring during the differentiation of DCs in psoriasis.

### 3.6 Identifying FABP5 and KLRB1 as robust markers in psoriasis via machine learning

**Figure 5:**
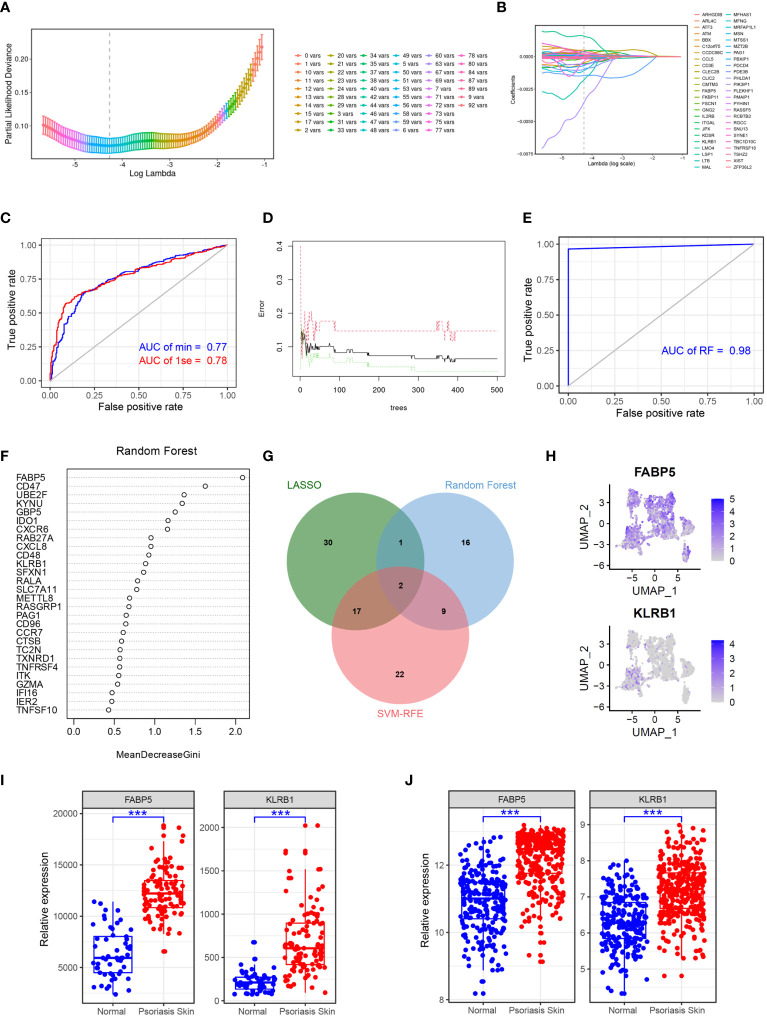
Identifying FABP5 and KLRB1 as robust markers in psoriasis via machine learning. **(A)** Cross-validation for parameter optimization classification model. **(B)** Construction of the LASSO regression model using GSE41664 samples as the training set. **(C)** ROC curve of LASSO regression classification model in GSE117468 validation set. **(D)** Construction of the random forest model using GSE41664 samples as the training set. **(E)** ROC curve of random forest classification model in GSE117468 validation set. **(F)** The key genes of psoriasis identified by random forest model. **(G)** Venn diagram showing the robust markers identified by LASSO, random forest, and SVM-RFE. **(H)** tSNE plot showing the expression of FABP5 and KLRB1 in DCs subclusters. **(I, J)** Box plot showing the expression of FABP5 and KLRB1 in normal and psoriasis skin tissues in GSE41664 and GSE117468, respectively. Significance levels: ***p < 0.001.

To identify key genes associated with psoriasis development, we employed a combination of the LASSO, random forest, and SVM-RFE algorithms. Using the LASSO algorithm, we identified 50 genes as robust markers in psoriasis (**Figures 5A, B**). The prediction performance of the LASSO Cox proportional hazard model, as assessed by ROC analysis, demonstrated relatively high accuracy (**Figure 5C**). The random forest algorithm further identified 28 genes significantly associated with psoriasis, including FABP5, CD47, and UBE2F (**Figures 5D-F**). FABP5 has been widely reported in autoimmune diseases such as multiple sclerosis, inflammatory neuronal remodeling, and DCs dysregulation (25–27). Similarly, CD47 and UBE2F are involved in aberrant functions of DCs and T cells in various tumors (28–30), indicating their potential relevance to psoriasis development. The random forest algorithm thus demonstrated high accuracy in predicting psoriasis. Furthermore, through the integration of the LASSO, random forest, and SVM-RFE algorithms, we identified FABP5 and KLRB1 as robust markers in psoriasis (**Figure 5G**). To validate these findings, we performed gene expression analysis of scRNA-seq data, which confirmed the overexpression of FABP5 and KLRB1 in cluster 1 DCs, reinforcing their potential role in psoriasis (**Figure 5H**). To further verify the predictive effect of FABP5 and KLRB1, we conducted differential expression analysis in two independent psoriasis cohorts. The results demonstrated that FABP5 and KLRB1 were significantly upregulated in psoriasis skin tissues (**Figures 5I, J**).

### 3.7 Clinical drug treatments change the composition of DCs

**Figure 6:**
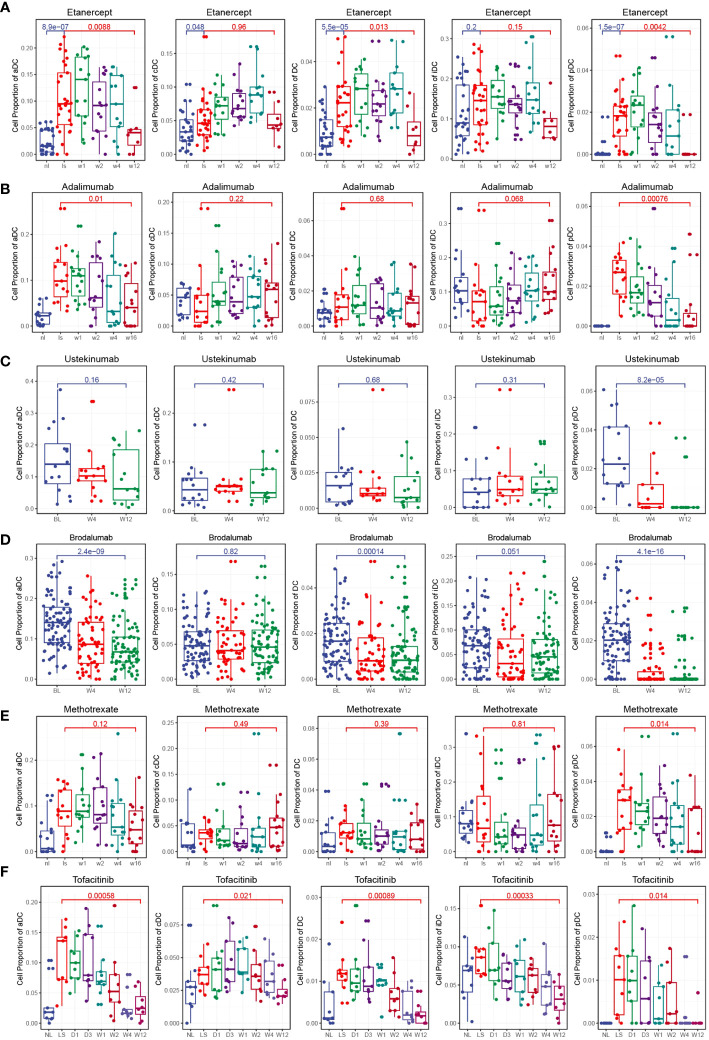
Clinical drug treatments change the composition of DCs. **(A)** Box plot showing the composition of DCs during etanercept treatment. Etanercept significantly decreased the composition of aDCs and pDCs. **(B)** Box plot showing the composition of DCs during adalimumab treatment. Adalimumab significantly decreased the composition of aDCs and pDCs. **(C)** Box plot showing the composition of DCs during ustekinumab treatment. Ustekinumab significantly decreased the composition of pDCs. **(D)** Box plot showing the composition of DCs during brodalumab treatment. Brodalumab significantly decreased the composition of aDCs and pDCs. **(E)** Box plot showing the composition of DCs during methotrexate treatment. Methotrexate treatment was found to inhibit pDCs. **(F)** Box plot showing the composition of DCs during tofacitinib treatment. Tofacitinib treatment demonstrated inhibition of multiple DC subsets, including total DCs, aDCs, cDCs, iDCs, and pDCs.

To investigate the effects of different clinical drug treatments on DCs in psoriasis patients, we performed immune infiltration analysis. The analysis included psoriasis patients treated with etanercept (GSE41664), brodalumab (GSE117468), ustekinumab (GSE117468), methotrexate (GSE85034), adalimumab (GSE85034), and tofacitinib (GSE69967). The results of the analysis revealed specific effects of certain drugs on the composition of DC subsets. Both etanercept and adalimumab significantly decreased the composition of activated DCs (aDCs) and plasmacytoid DCs (pDCs) (**Figures 6A, B**). This suggests that the specific inhibition of TNF-α, which is the target of these drugs, leads to a reduction in aDCs and pDCs, thereby relieving the symptoms of psoriasis. Ustekinumab and brodalumab significantly decreased the composition of pDCs (**Figures 6C, D**). This indicates that the targeted blocking of IL-12 and IL-17, respectively, inhibits the differentiation of pDCs, thus contributing to the therapeutic effect in psoriasis. Methotrexate treatment was found to inhibit pDCs (**Figure 6E**). This suggests that methotrexate has a specific suppressive effect on pDCs in psoriasis patients. Tofacitinib treatment demonstrated inhibition of multiple DC subsets, including total DCs, aDCs, conventional DCs (cDCs), immature DCs (iDCs), and pDCs (**Figure 6F**). This broad inhibition indicates that tofacitinib affects various DC populations, potentially contributing to its therapeutic effect in psoriasis. Overall, the immune infiltration analysis of psoriasis patients treated with different drugs revealed distinct effects on DC subsets. These findings provide insights into the mechanisms of action of these drugs and their specific targeting of DC populations in the context of psoriasis treatment.

### 3.8 FABP5 and KLRB1 were associated with the fraction of T cell during clinical treatments

**Figure 7:**
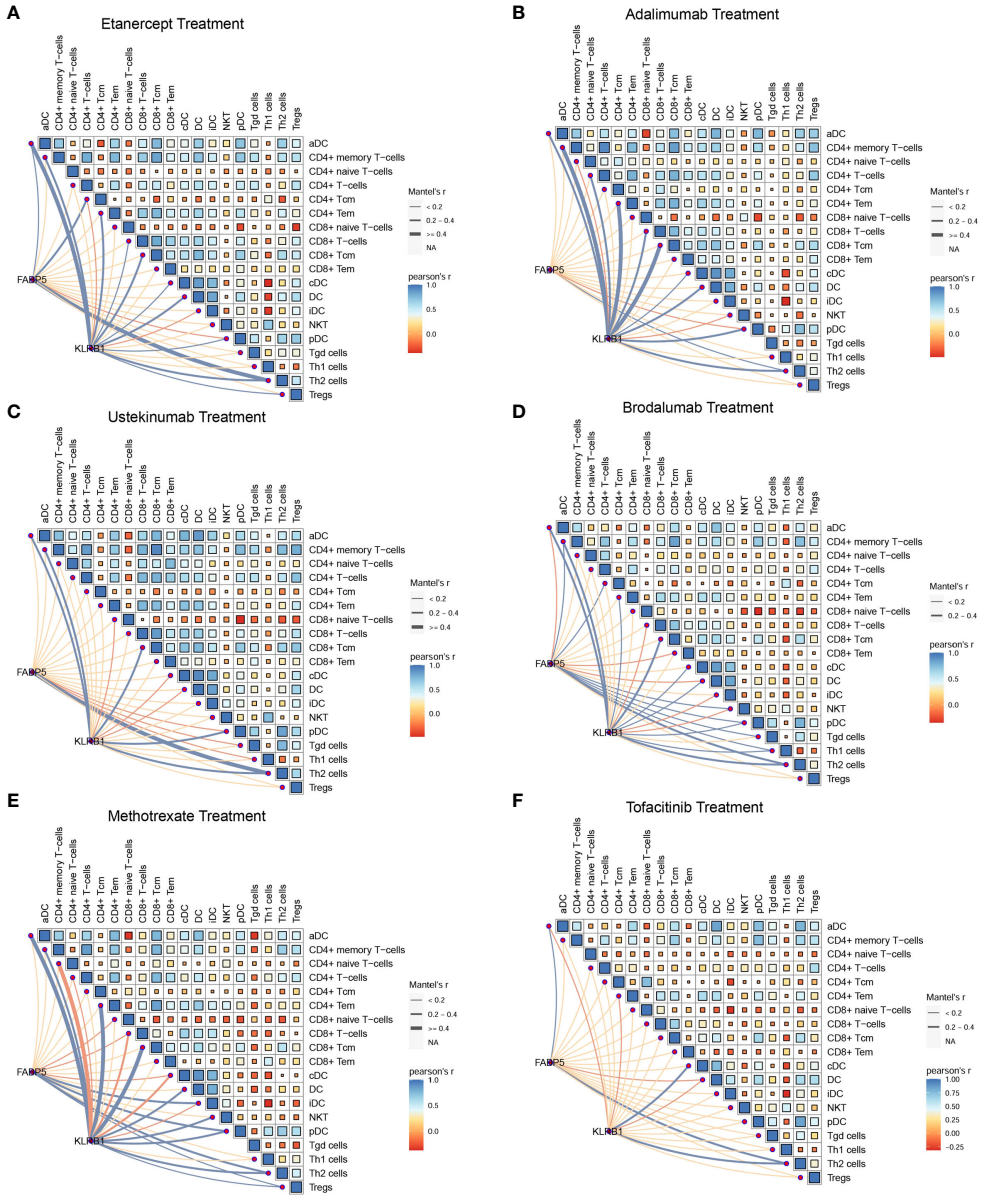
FABP5 and KLRB1 were associated with the fraction of T cell during clinical treatments. **(A, B)** The correlation between the expression of FABP5, KLRB1 and the composition of different T cells during etanercept and adalimumab treatments. FABP5 expression showed a significant correlation with CD4+ Tcm and Th2 cells, while KLRB1 expression was associated with the activation of CD4+ T Tem, CD8+ Tcm, and Th2 cells. **(C, D)** The correlation between the expression of FABP5, KLRB1 and the composition of different T cells during ustekinumab and brodalumab treatments. FABP5 expression demonstrated a correlation with Th2 cells, whereas KLRB1 expression was associated with CD8+ Tcm and Th2 cells. **(E, F)** The correlation between the expression of FABP5, KLRB1 and the composition of different T cells during methotrexate and tofacitinib treatments. FABP5 and KLRB1 expressions were associated with Th2 cells.

To investigate the relationship between FABP5, KLRB1, and T cells in psoriasis, we performed Pearson’s correlation analysis using data from a psoriasis cohort. The results of the correlation analysis revealed associations between the expression of FABP5 and KLRB1 and specific T cell subpopulations in different treatment conditions. For etanercept and adalimumab treatments, FABP5 expression showed a significant correlation with CD4+ T central memory cells (Tcm) and Th2 cells, while KLRB1 expression was associated with the activation of CD4+ T effector memory cells (Tem), CD8+ Tcm, and Th2 cells (**Figures 7A, B**). For ustekinumab and brodalumab treatments, FABP5 expression demonstrated a correlation with Th2 cells, whereas KLRB1 expression was associated with CD8+ Tcm and Th2 cells (**Figures 7C, D**). For methotrexate and tofacitinib treatments, both FABP5 and KLRB1 expressions were associated with Th2 cells (**Figures 7E, F**). These findings suggest specific associations between FABP5 and KLRB1 expressions and particular T cell subpopulations in the context of different treatments for psoriasis. The correlation analysis indicates potential roles of FABP5 and KLRB1 in modulating immune responses mediated by CD4+ Tcm, CD8+ Tcm, and Th2 cells during the respective treatment regimens. These correlations provide insights into the interactions between FABP5, KLRB1, and T cells, contributing to our understanding of the underlying immunological mechanisms in psoriasis and the effects of different therapeutic interventions.

### 3.9 FABP5 and KLRB1 are highly expressed in psoriatic human skin tissues and M5-induced HaCaT cells

**Figure 8:**
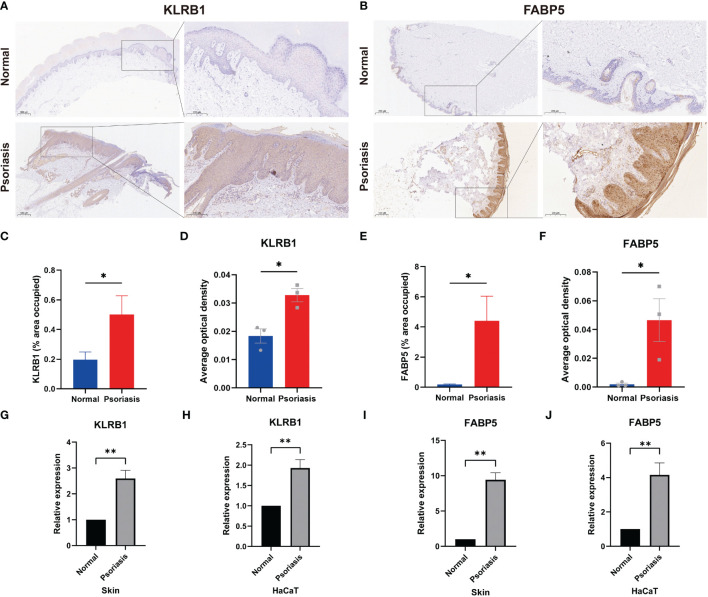
Expression of FABP5 and KLRB1 in human skin tissue and HaCaT cells. **(A, B)** Immunohistochemical (IHC) staining demonstrates FABP5 and KLRB1 expression in the skin of psoriasis patients (n=3) and healthy controls (n=3). Full-size and zoom-in images were acquired at 5x and 20x. **(C, E)** Quantification of IHC images is represented as the percentage of occupied area ± SEM. **(D, F)** Quantification of IHC images is represented as the average optical density ± SEM. **(G-J)** Relative mRNA expression of FABP5 and KLRB1 in the skin of psoriasis patients and psoriasis model *in vitro* by RT-qPCR. (*P<0.05; **P<0.01).

To validate the results of single-cell and machine learning analyses, we conducted experimental validation through immunohistochemistry, RT-qPCR, and immunofluorescence. In psoriasis patients, KLRB1 exhibited heightened expression in the stratum corneum and dermis, while FABP5 showed increased expression in the entire dermis and epidermis, as indicated by brownish-yellow coloration in corresponding immunohistochemistry images (**Figures 8A, B**). Semi-quantitative analysis revealed an average positively stained area of 0.5016 for KLRB1 in the psoriasis group, compared to 0.1971 in the normal group. Conversely, FABP5 averaged 4.409 in psoriasis and only 0.1871 in the normal group. AOD values for KLRB1 and FABP5 in psoriatic skin tissues were 0.03278 and 0.04656, respectively, versus 0.01833 and 0.001889 in normal tissues (**Figures 8C-F**). RT-qPCR results demonstrated a 2.595-fold increase in KLRB1 and a 9.418-fold increase in FABP5 expression in psoriatic skin tissues compared to the control group. Moreover, KLRB1 and FABP5 expression in HaCaT cells, an *in vitro* model of psoriasis induced by M5, was 1.93-fold and 4.158-fold higher than the control group, respectively (**Figures 8G-J**). The substantial evidence suggests an elevation in FABP5 and KLRB1 expression in psoriasis, with a more pronounced trend in FABP5 than in KLRB1.

### 3.10 Immunofluorescence reveals co-localization of KLRB1 and FABP5 in human psoriasis skin tissues

**Figure 9:**
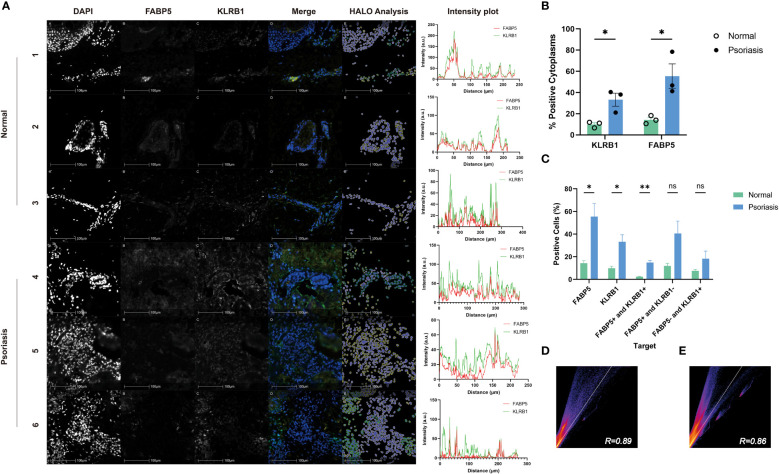
Immunofluorescence analysis of FABP5 and KLRB1 in human skin tissues. **(A)** Representative images of fluorescence immunohistochemistry for FABP5 and KLRB1. Numbers 1-3 are normal human skin from different individuals, 4-6 are from psoriasis patients. Digital image analysis utilizing HALO to quantify the percentage of FABP5 and KLRB1 positive cells. Each Intensity plot demonstrates the colocalization analysis of FABP5 with KLRB1 for different groups. **(B)** The percentage of positive cytoplasm increased for both KLRB1 and FABP5 within the psoriasis group by HALO analysis. **(C)** The percentage of positively stained cells for KLRB1 and FABP5(single-positive, double-positive, and double-negative instances) by HALO analysis. **(D, E)** Colocalization analysis for images 1-3 **(D)** and 4-6 **(E)** by Image J. Data are presented as mean ± SEM. Significance levels: * p < 0.05, ** p < 0.01, and ns indicates non-significance.

To explore the correlation between FABP5 and KLRB1, we examined skin lesions from three psoriasis patients and three healthy controls using immunofluorescence double staining. In the Merge plot, blue represents DAPI-stained nuclei, green indicates KLRB1, and yellow denotes FABP5. The psoriasis group exhibited more pronounced green and yellow fluorescence in the images. For analysis, we employed the HALO platform, marking FABP5-positive cells with yellow circles, KLRB1-positive cells with green circles, and negative cells with white circles (**Figure 9A**). Quantitative data from HALO revealed significantly higher mean values of positive cytoplasmic percentage for KLRB1 (33.24) and FABP5 (55.47) in the psoriasis group compared to the control group (9.932 and 14.30, respectively) (**Figure 9B**). Quantitative analysis further demonstrated a statistically significant elevation in FABP5 positivity (55.47 *vs*. 33.24) and KLRB1 positivity (14.30 *vs*. 9.932) in the psoriasis group (p-values 0.0253 and 0.0211, respectively). Double positivity for FABP5 and KLRB1 was 14.91 in the psoriasis group and 2.389 in the control group, exhibiting a slightly lower yet statistically more significant tendency for elevation than the first two groups (p-value 0.0024). FABP5 positivity coupled with KLRB1 negativity was observed at 40.56 in the psoriasis group and 11.91 in the normal group, while KLRB1 positivity with FABP5 negativity stood at 18.33 in the psoriasis group and 7.542 in the normal group. Despite these numerical differences between psoriasis and normal groups, no statistically significant distinction emerged (**Figure 9C**). The intensity plot reveals a synchronized trend in fluorescence intensity profiles of both staining channels, demonstrating convergence in the fluorescence of FABP5 and KLRB1 at the same position. The corresponding Pearson’s correlation coefficients for the psoriasis and control groups were 0.89 and 0.86, respectively (**Figures 9D, E**). Combined with the analysis of fluorescence intensity curves, we deduce a co-localization of KLRB1 and FABP5 in both psoriasis and normal skin tissues, elucidating the characteristics of KLRB1 and FABP5 in these contexts.

## 4 Discussions

We looked at the specific immune microenvironment of clinical relevance and screened for new key genes in diagnosis and new targets in therapy based on data obtained from single-cell RNA sequencing analysis of 13 psoriatic skin tissues versus 5 normal control skin tissues in the original literature and information obtained from the GSE dataset (31). We analyzed KCs, melanocytes, T cells, macrophages, and DCs in psoriasis lesions. We identified unique expression profiles of KCs and DCs, further explored the importance of altered DC function in the development and progression of psoriasis, and identified two key genes, FABP5 and KLRB1, from the initially predicted 50 genes by a machine learning approach. Our results highlight the potential molecular mechanisms and novel biomarkers for psoriasis. At the same time, the specific targeting of DCs by different drugs observed in therapy could provide more powerful information for better treatment of gene-driven autoimmune diseases.

This study investigates the aberrant function of DCs in psoriasis pathogenesis. DCs possess the ability to acquire, process, retain, and present peptides on major histocompatibility complex (MHC) molecules, crucial for regulating adaptive immunity and activating auto-reactive pathogenic T cells (32), which cross-present exogenous soluble cell-associated antigens to CD8+ T cells and regulate histopathology through multiple mechanisms, including pathogenic and regulatory T cell initiation and differentiation, local reactivation of autoimmune T cells within target tissues, and control of epitope spreading, so that the development of many autoimmune diseases is associated with genetics and variation in DC function (33–36). Central to the pathogenesis of psoriasis is the cross-talk between the innate and adaptive T cell-based immune systems involving mainly DCs, macrophages, mast cells, and granulocytes, as well as the effects of the immune response on and interactions between various cellular subpopulations in the skin (37). DCs serve as both initiators of antigen presentation and orchestrators of inflammatory pathways in psoriasis. PDCs induce mDCs to release pro-inflammatory mediators, establishing type I and type II IFN inflammatory cascades in collaboration with IFN-γ-producing T cells (Tc1 and Th1), mediated by CXCL10 and CXCL9 feedback mechanisms (1). Inflammatory myeloid DCs and mature dermal DCs produce cytokines such as IL-12 and IL-23 to activate Th1, Th17, Th22, and Tc17 cells, which in turn act on KCs (38). ADCs migrate to the draining lymph nodes, secreting inflammatory factors like IL-23, TNF-α, IL-1β, and IL-12. Single-cell analysis showed increased expression of CD5-CD163+CD14+ DCs in psoriatic lesion skin and production of IL23A and IL1B (39). CD4+ naïve T cells, stimulated with IL-12, differentiate into Th1 cells, secreting IL-23 to support the survival and proliferation of Th17 and Th22 cells. Other inflammatory cells co-produce IL-17, IL-22, and IL-12 with T cells. This cascade leads to downstream proliferation of KCs, heightened expression of inflammatory mediators, endothelial adhesion molecules, and immune cell infiltration. The skin inflammation in psoriasis is predominantly caused by pathogenic cytokines produced by overly dysregulated T cells (40, 41). Our findings are consistent with the existing views and reaffirm the interaction between DCs and T cells in psoriasis. The finding that the CD70/CD27 signaling pathway serves as an important link between the two provides a new theoretical basis for unraveling the molecular mechanisms of DCs in psoriasis.

The CCR7/CCL19 signaling pathway is an important signaling pathway present in psoriasis, and studies demonstrate that inhibiting TNF leads to suppression of this pathway, resulting in dispersal of lymphoid clusters marked by the activation of this pathway, leading to amelioration of psoriasis symptoms (42, 43). This provides evidence that TNF-α inhibitors, represented by etanercept and adalimumab, can treat psoriasis by altering the composition of DCs. CCR7 is a pivotal chemokine receptor in immune function driven by lymphocytes and is expressed across diverse immune cell subpopulations, with CCL19 and CCL21 as its ligands (44). Prior research indicates that CCL19 is selectively synthesized near perivascular T cells and DCs, potentially facilitating the recruitment of CCR7+ T cells and DCs to these sites (43). DCs can enhance their responsiveness to CCL19 and CCL21 by upregulating CCR7 expression, and additionally modulate T cells via their production of CCL19 (45). DCs and T cells utilize the CCR7/CCL19 axis as a conduit in influencing the pathogenesis of psoriasis via interactions within psoriatic dermal aggregates. These studies have augmented the understanding of DC involvement in psoriasis at the molecular level and furnish crucial theoretical underpinnings for the findings presented herein.

However, our current study underscores the significant role of DCs in psoriatic skin by orchestrating T cell regulation through the CD70/CD27 signaling pathway. CD70, belonging to the TNF superfamily and acting as a ligand for CD27, exhibits predominant expression on aDCs but is also present on Th1 and Th17 cells. CD27 signaling suppresses the expression of Th17-associated genes in regulatory T cells (Tregs) (46, 47). The CD70/CD27 signaling pathway is mainly regulated by CD70 expression, which directly regulates T cell-T cell interactions and influences the development of effector T cells. The high expression of CD70 can directly High CD70 expression can directly induce CD27+ lymphocyte over-infiltration, while CD27 and CD70 co-stimulation of the pathway can impede Th17 effector cell differentiation and associated autoimmunity (48). CD27 signaling does not affect master regulators of T helper cell lineage commitment. However, it selectively inhibits the psoriasis-associated pathogenic factor IL-17 and chemokine receptor CCR6 in differentiated Th17 cells through the c-Jun N-terminal kinase (JNK) pathway transcription in differentiated Th17 cells (49). CD27 serves as a distinguishing marker for γδ T cell subsets based on their cytokine profiles, delineating IFN-γ producers as CD27+ and IL-17 producers as CD27-CCR6+ (50). IL-1β induces keratinocytes to secrete chemokines, specifically attracting CD27-CCR6+ γδ T cells (51). CD19(+)CD27(+)CD24(high) Breg cells exhibit reduced expression in psoriasis and psoriatic arthritis patients, correlating inversely with Psoriasis Area and Severity Index (PASI) scores (52). Moreover, psoriasis patients demonstrate reduced expression of CD27 and CD28 on skin T cells (53). Patients with psoriasis exhibit a decreased frequency of CD27(+) memory B cells, contrary to observations in atopic dermatitis (AD), which has a predominantly Th2 response (54). CD27+ Tregs can inhibit the expression of their co-stimulatory molecule CD70 on the plasma membrane of DCs. Down-regulation of CD70 necessitates contact between Tregs and DCs facilitating the endocytosis of CD27 and CD70 by the DCs. This mechanism enables Tregs to sustain tolerance or inhibit excessive proinflammatory Th1 responses (55, 56). During the triggering phase of allergic contact dermatitis, there is an increase in the expression of CD70 and the Th17-specific transcription factor retinoid orphan receptor gamma T. Managing this phenomenon could ameliorate symptoms of other autoimmune diseases like psoriasis. Additionally, activated NKT cells can induce the expression of CD70 on DCs (57). Therefore, we hypothesized that DCs are involved in the pathogenesis of psoriasis by regulating T cells via the CD70/CD27 signaling pathway.

FABP5, also known as epidermal-FABP (E-FABP) (58), was first identified due to its significant up-regulation in psoriatic KCs (59). FABP5 is highly expressed in epidermal cells and is also present in brain, kidneys, liver, lungs, testes, adipose tissue and macrophages (60–62). It is crucial to maintain lipid and glucose homeostasis and regulate insulin responses and inflammation (63). Studies on cardiometabolic risk suggest an association between FABP5 and the development of obesity-related metabolic syndrome (MetS) and atherosclerosis (64). Consequently, FABP5 may link psoriasis and MetS, although further investigation is required to investigate the underlying mechanisms. FABP5 is strongly expressed in human cutaneous CD8+ TRM cells and is critical for the long-term persistence of this cell in the skin. T-cell-specific deficiency of Fabp5 impairs the uptake of exogenous free fatty acid by CD8+ TRM cells and reduces the long-term viability of CD8+ TRM cells *in vivo*. Also, cutaneous CD8+ TRM cells lacking Fabp4/Fabp5 in a mouse model were significantly reduced in their ability to resist cutaneous viral infections. Psoriasis, a disease that can be mediated by CD8+ TRM cells co-expressing CD8 and CD69, has detectable FABP5 protein expression in skin lesions. We hypothesize that FABP5 may play a role in the pathogenesis of psoriasis by affecting lipid metabolism and causing TRM cell dysfunction (65–67). Additionally, FABP5 might regulate the differentiation of psoriatic KCs through the NF-κB signaling pathway (68, 69). Previous studies have correlated serum FABP5 with both the PASI score and the basic inflammatory index in psoriasis patients, indicating its potential as a marker for psoriasis severity and clinical outcomes post-treatment with NB-UVB (70, 71). However, Tomomi Miyake et al. concluded that FABP5 was not associated with PASI scores and that there was no significant, consistent increase or decrease in FABP5 levels after treatment with Adalimumab, Infliximab, and NB-UVB (72). Further studies are essential to reconcile these discrepancies. FABP5 expression has been linked to the activation of various pathways, including NLRP3/IL-1β, RAR/CD11c, LTA4/LTB4, or STAT1/2/IFNβ (58). The novel topical drug VX-509 also has shown efficacy in diminishing psoriasis inflammation by targeting the STAT3/FABP5 pathway (73).

The gene KLRB1 encodes the cell surface molecule CD161 (74), which is expressed in T cells, natural killer (NK) cells, and various lymphocyte populations in the thymus, cord blood, and peripheral blood. Numerous studies have illustrated the association of KLRB1 expression with the prognosis of various cancers (75). Additionally, CD161-expressing T cells exhibit highly functional pro-inflammatory characteristics, contributing to autoimmune disease development (76, 77). NK cells, a class of innate lymphocyte populations capable of distinguishing between infected or stressed cells and healthy cells (78), have been found to produce the psoriasis-causing cytokines IL-17 and IL-22 (79). CD161 is early in its expression during NK cell development and may facilitate crosstalk between NK cell precursors and bone marrow cells, leading to CXCL8 release. Moreover, in psoriatic lesions, the upregulated expression of CD1d in KCs activates NKT cells, leading to increased production of the pathogenic factor IFN-γ, thereby promoting psoriasis progression (80, 81). The primary functional disparity between CD161+ and CD161- NK cells lies in their responsiveness to pro-inflammatory cytokines. CD161 serves as a marker for NK cells that can respond to IL-12 and IL-18 during differentiation, leading to the subsequent upregulation of NKp30, CD160, CD25, CD69, and IFN-γ—an identified psoriasis-associated pathogenic factor (82). Research has shown that a subset of CD4 T memory cells, both circulating and residing in the intestines, expressing CD161, can respond to IL-23. This response leads to the full differentiation and activation of Th17 cells, underscoring the significance of this subpopulation in the inflammatory response (83).

FABP5 and KLRB1 have been previously studied in relation to other dermatologic conditions. FABP5 is elevated in patients with AD, especially in combination with atopic march, and correlates with the severity of skin involvement (84, 85). Up-regulated FABP5 is implicated in radiation-induced skin fibrosis via modulation of the TGF-β signaling pathway. Proteomic profiling revealed heightened expression of KRT6C and FABP5 in arsenic-induced keratosis pilaris (86, 87). Additionally, FABP5 plays a significant role in skin tumors; suppressing FABP5 or S100A9 expression impedes the proliferation and migration of cutaneous squamous cell carcinoma cells via the NF-κB pathway (88). Within extramammary Paget’s disease tumor tissues, FABP5 exhibits co-localization with CK7, CK20, and EMA, accompanied by substantial expression levels (89). Similarly, mycosis fungoides manifests elevated expression levels of FABP5, S100A8, and SOD2. The study of KLRB1 in dermatology has been limited. Still, single-cell analysis of skin tissues and peripheral blood mononuclear cells from Palmoplantar pustulosis, a specific psoriasis subtype, revealed heightened expression and co-expression of TH17 (KLRB1/CD161) and TH2 (GATA3) in a subset of memory CD4+ T cells. Memory CD4+ T cell subsets labeled with TH17 demonstrated plasticity towards TH2, with high expression and co-expression of CD161 and GATA3 observed in skin lesions (90). FABP5 is linked to the fatty acid-rich microenvironment of the skin, and its elevation is common in various dermatologic conditions. While KLRB1 and FABP5 have been noted in other dermatologic conditions, their precise molecular mechanisms and disease risks remain unclear. FABP5 alone lacks specificity as a biomarker for psoriasis, but its combination with KLRB1 enables differentiation from other skin diseases. Thus, FABP5 and KLRB1 hold promise as significant biomarkers for psoriasis diagnosis. The downstream pathways of FABP5 vary across various dermatologic diseases, and our team’s future research will concentrate on the detailed mechanistic examination of FABP5 and KLRB1 in psoriasis.

In this study, we evaluated six commonly prescribed psoriasis treatments, and overall, tofacitinib emerges as the preeminent choice for targeting DCs in psoriasis treatment. As a first-generation Janus kinase inhibitor, tofacitinib is the initial oral JAK approved in the European Union and the United States (91–93), demonstrating superior efficacy in clinical trials. Currently, these inhibitors are in use for treating rheumatoid arthritis, psoriatic arthritis, and ulcerative colitis, demonstrating significant efficacy and safety in clinical trials (94, 95). Tofacitinib inhibits kinases such as JAK1, JAK2, and JAK3, along with cytokines such as γ-chain cytokines, IFN-γ, and IL-6 (96). Additionally, *in vitro* studies reveal its inhibition of the signaling process of IFN-α/β and IL-22 in isolated KCs (97). While several clinical trials affirm its effectiveness in psoriasis by suppressing various psoriasis-related cytokines (98–101), the U.S. Food and Drug Administration, the European Medicines Agency, and China have not yet added psoriasis to the indications for tofacitinib. Despite scarce clinical data, our study’s observation of tofacitinib significantly reducing DCs in psoriasis suggests its potential as an efficacious psoriasis treatment. Our immunohistochemistry, PCR, and immunofluorescence experimental validation demonstrated the upregulation and co-localization of FABP5 and KLRB1 in human psoriatic skin tissues and M5-induced HaCaT cells. The lack of significance in the percentage of cells positive for either KLRB1 or FABP5, while concurrently being negative for the other, could stem from the limited number of studies and potential errors. However, combined with the analysis results in the previous part of this paper, this may also be a conclusion in line with the objective facts. The double positive of FABP5 and KLRB1 has higher specificity in the psoriasis group. The elevation of FABP5 in the psoriasis group is more pronounced. Conversely, negativity for either FABP5 or KLRB1 could indicate a protective factor against psoriasis. Additional investigations are needed to explore the relationship and mechanisms of action between FABP5 and KLRB1 in psoriasis.

Our study delved into the mechanisms underlying DCs-T cell interactions in psoriasis pathogenesis, identifying novel biomarkers and molecular targets for diagnosis and treatment. We hope our discoveries offer fresh insights into psoriasis diagnosis and therapeutic strategies. Nevertheless, our study has limitations; firstly, relying on bioinformatics analysis introduces potential bias due to limited data and varying statistical methods. Secondly, the sample size for RT-qPCR and immunohistochemistry might need to be increased. Hence, further research must substantiate our findings with more robust evidence.

## 5 Conclusion

We explored psoriasis’s pathogenesis and diagnostic biomarkers through advanced bioinformatics methods, including single-cell sequencing data analysis and machine learning. Findings were validated using RT-qPCR, immunohistochemistry, and immunofluorescence. Our data indicate that DCs, particularly through the CD70/CD27 pathway, mediate inflammatory responses in psoriasis by influencing other cells, especially T cells. Additionally, FABP5 and KLRB1 emerge as potential therapeutic targets, exhibiting correlation with specific T-cell subsets during treatment. Tofacitinib demonstrates advantages in studying drugs targeting DCs, with elevated expression and co-localization of FABP5 and KLRB1 in psoriasis. These insights enhance our understanding of psoriasis, elucidate the connection between key genes and the immunoregulatory network, and pave the way for novel diagnostic and therapeutic strategies.

## Data availability statement

Publicly available datasets were analyzed in this study. This data can be found here: GSE151177 (https://www.ncbi.nlm.nih.gov/geo/query/acc.cgi?acc=GSE151177), GSE41664 (https://www.ncbi.nlm.nih.gov/geo/query/acc.cgi?acc=GSE41664), GSE85034 (https://www.ncbi.nlm.nih.gov/geo/query/acc.cgi?acc=GSE85034), GSE117468 (https://www.ncbi.nlm.nih.gov/geo/query/acc.cgi?acc=GSE117468) and GSE69967 (https://www.ncbi.nlm.nih.gov/geo/query/acc.cgi?acc=GSE69967).

## Ethics statement

The studies involving humans were approved by Medical Ethics Committee of the Second Affiliated Hospital of Harbin Medical University. The studies were conducted in accordance with the local legislation and institutional requirements. The participants provided their written informed consent to participate in this study.

## Author contributions

ZM: Conceptualization, Data curation, Investigation, Validation, Visualization, Writing – original draft, Writing – review & editing. PA: Conceptualization, Data curation, Investigation, Methodology, Writing – original draft, Writing – review & editing. SH: Funding acquisition, Validation, Writing – review & editing. ZH: Data curation, Writing – review & editing. AY: Resources, Writing – review & editing. YL: Supervision, Writing – review & editing. JT: Funding acquisition, Resources, Writing – review & editing.
