# Supplementary material for: Single-cell sequencing analysis and multiple machine-learning models revealed the cellular crosstalk of dendritic cells and identified FABP5 and KLRB1 as novel biomarkers for psoriasis
Source: Front Immunol. 2024 Mar 26;15:1374763. doi: 10.3389/fimmu.2024.1374763 (PMC11002082; doi:10.3389/fimmu.2024.1374763)
Supplement: Supplementary file 1 [file DataSheet_1.docx]

Supplementary Material

Single-cell sequencing analysis and multiple machine-learning models revealed the cellular crosstalk of dendritic cells and identified FABP5 and KLRB1 as novel biomarkers for psoriasis

# Supplementary Figures
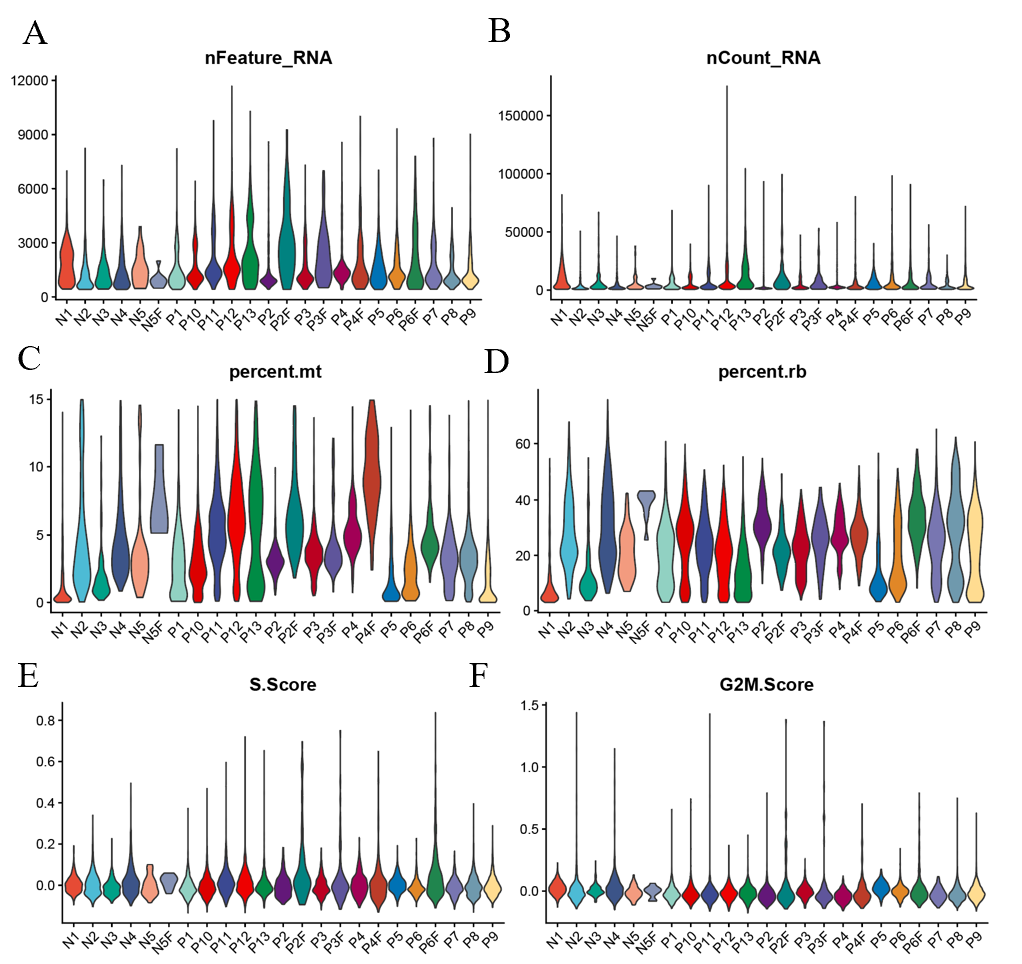


**Supplementary Figure 1.** **The standard of quality control in scRNA-seq analysis processing.** (A) Violin plot showing the range of features in different samples. (B) Violin plot showing the range of counts in different samples. (C) Violin plot showing the ratio of mitochondrial genes in different samples. (D) Violin plot showing the ratio of ribosomal genes in different samples. (E) Violin plot showing the S stage score in different samples. (F) Violin plot showing the G2M stage score in different samples.


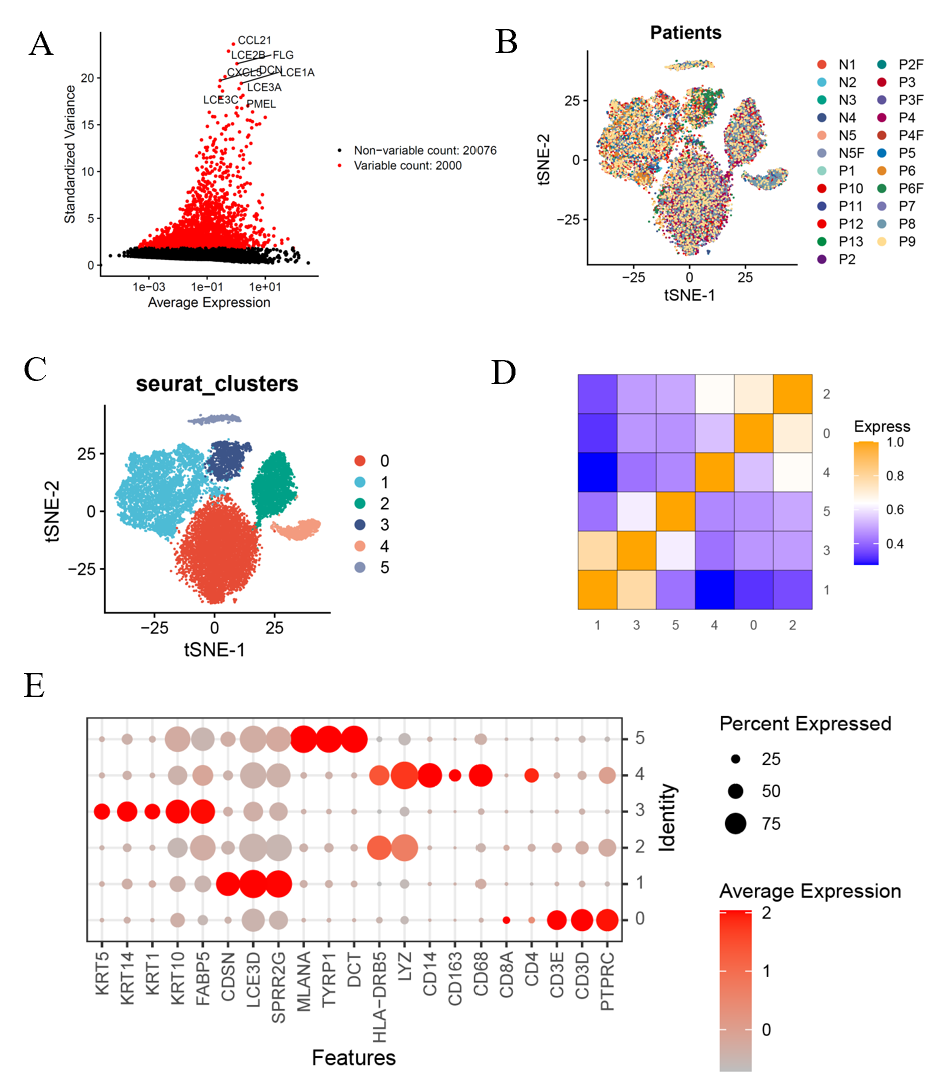


**Supplementary Figure 2.** **The process of cell type annotation in scRNA-seq analysis.** (A) Scatter plot showing the 2000 hypervariable genes in 20076 cells. (B) tSNE plot showing the 20076 cells extracted from 5 normal skin tissues and 13 psoriasis skin tissues. (C) tSNE plot showing the 20076 cells in 6 distinct psoriasis cell type clusters with K-means algorithm. (D) Heatmap showing the correlation among 6 psoriasis cell type clusters. (E) Bubble plot depicting expression of universal marker genes and percentage of positive cells in different clusters.


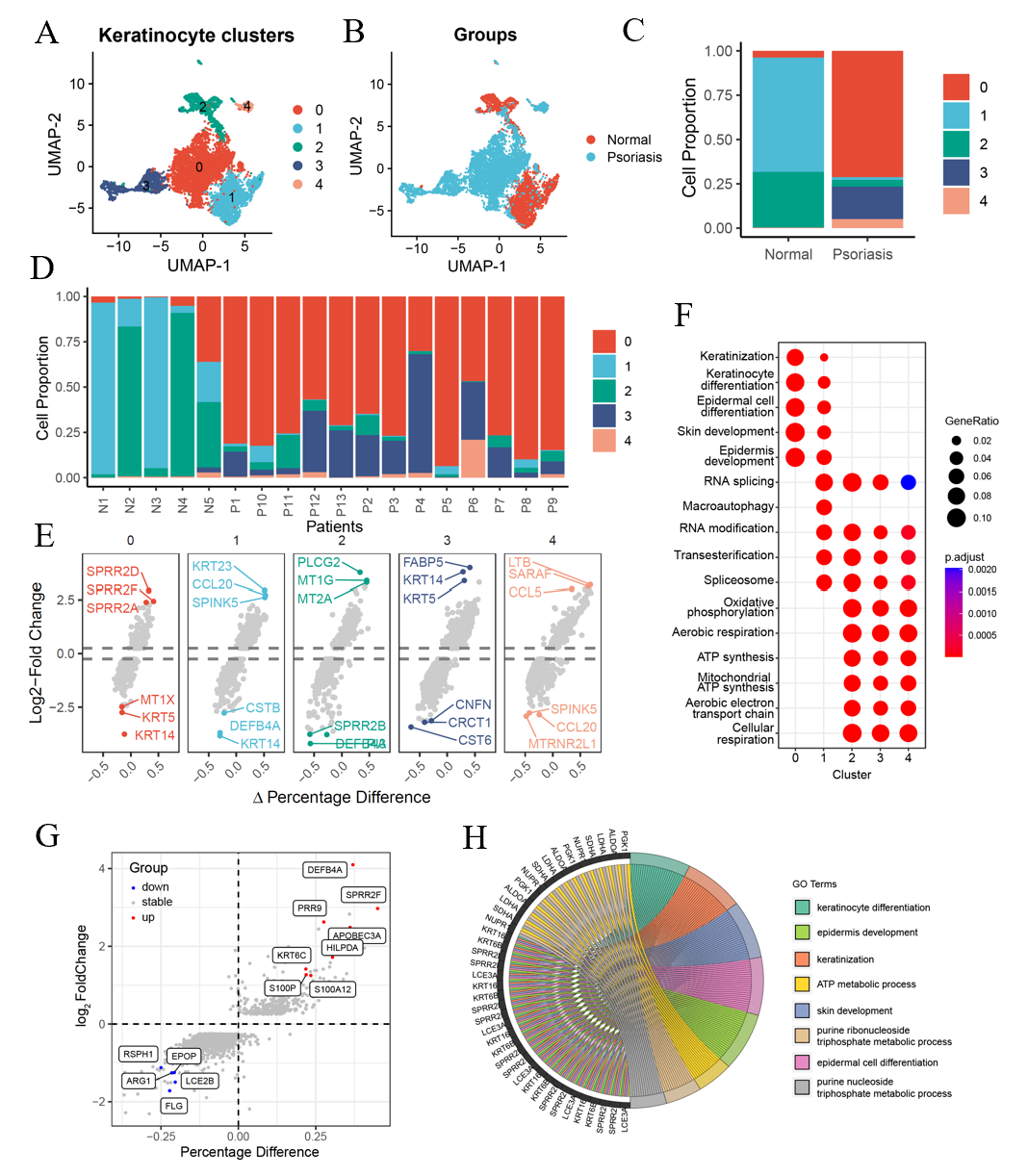


**Supplementary Figure 3.** **RNA splicing pathways were inactivated in keratinocytes from psoriasis skins.** (A) tSNE plot showing the 8326 keratinocytes in 5 distinct clusters with K-means algorithm. (B) tSNE plot showing the 8326 keratinocytes extracted from normal and psoriasis skin groups. (C) Bar plot showing the keratinocytes proportion in normal and psoriasis groups. (D) Bar plot showing the keratinocytes proportion in 5 normal skin tissues and 13 psoriasis skin tissues. (E) Volcano plot showing the highly expressed genes in 5 distinct keratinocytes clusters. (F) Dot plot showing the signaling pathways enriched in distinct keratinocytes clusters. (G) Volcano plot showing DEGs of keratinocytes between psoriasis and normal skin tissues. (H) Chord diagram showing the alternative pathways of keratinocytes between psoriasis and normal skin tissues.


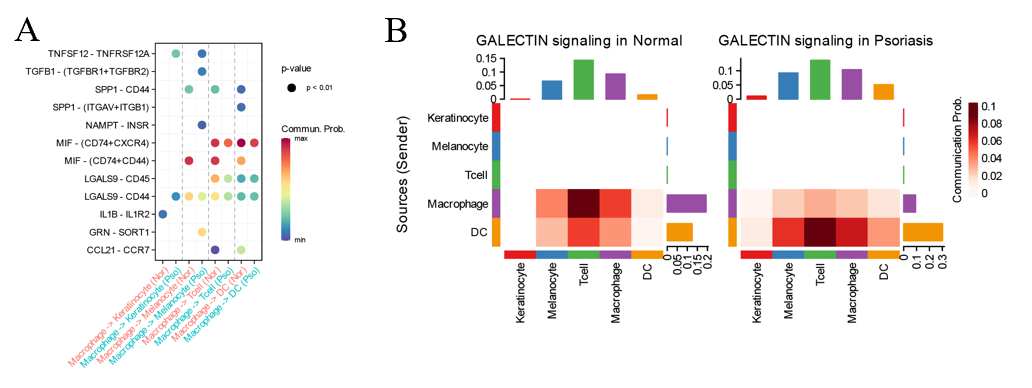


**Supplementary Figure 4.** **Regulation of T cells by macrophages.** (A) Dot plot showing the potential mechanism of cell-cell communications from macrophages to other cell types. (B) Heat map showing the differential number of interactions and differential n interaction strength between normal tissues and psoriasis tissues.


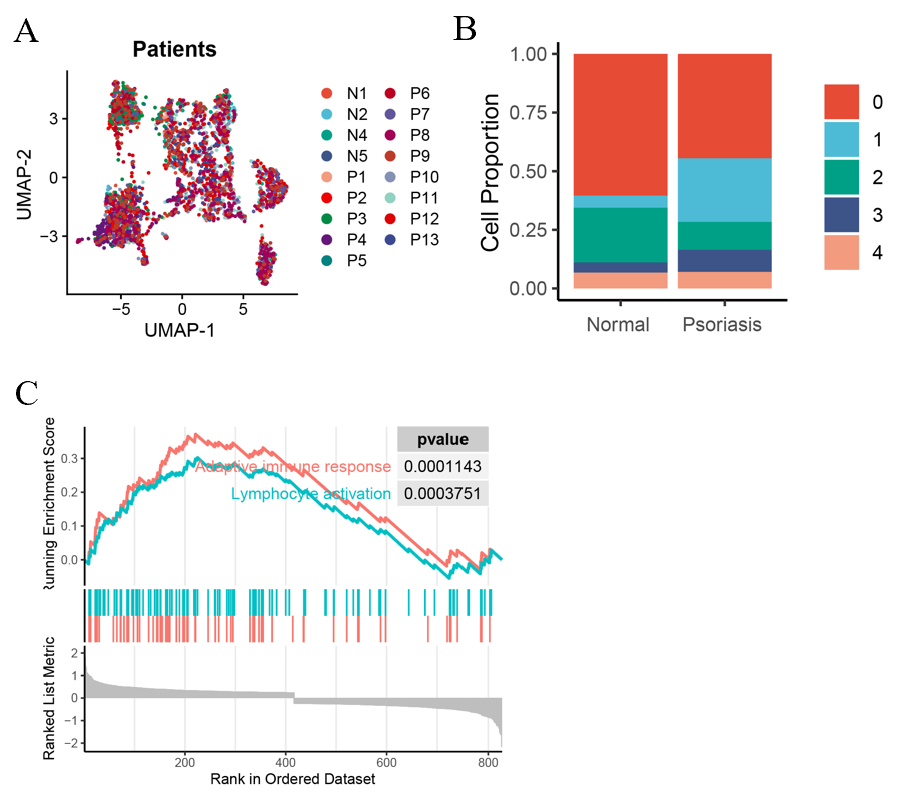


**Supplementary Figure 5.** **Inflammation-related pathways were significantly activated in DCs extracted from psoriasis.** (A) tSNE plot showing the 2988 cells extracted from 4 normal skin tissues and 13 psoriasis skin tissues. (B) Bar plot showing the DCs proportion in normal and psoriasis groups. (C) GESA results showing the activated signaling pathways in DCs extracted from psoriasis skin tissues.

# Supplementary Tables

| NO. | Cohort ID | Samples | Type | Source website |
| --- | --- | --- | --- | --- |
| 1 | GSE151177 | 23 | Expression profiling by high throughput sequencing | https://www.ncbi.nlm.nih.gov/geo/query/acc.cgi?acc=GSE151177 |
| 2 | GSE41664 | 157 | Expression profiling by array | https://www.ncbi.nlm.nih.gov/geo/query/acc.cgi?acc=GSE41664 |
| 3 | GSE85034 | 179 | Expression profiling by array | https://www.ncbi.nlm.nih.gov/geo/query/acc.cgi?acc=GSE85034 |
| 4 | GSE117468 | 565 | Expression profiling by array | https://www.ncbi.nlm.nih.gov/geo/query/acc.cgi?acc=GSE117468 |
| 5 | GSE69967 | 95 | Expression profiling by array | https://www.ncbi.nlm.nih.gov/geo/query/acc.cgi?acc=GSE69967 |

**Table S1. The information of cohorts used in this study**

| Primer name | Sequence |
| --- | --- |
| FABP5(F) | TGGCCAAGCCAGATTGTATCA |
| FABP5(R) | CTGATGCTGAACCAATGCACC |
| KLRB1(F) | AATTTGCCCTGAAACTTAGCTG |
| KLRB1(R) | GGATGTCACTGAAACACTCAAC |
| GAPDH(F) | CTGCCACCCAGAAGACTGTG |
| GAPDH(R) | CAGCTCAGGGATGACCTTGC |

**Table S2. The information of primers used in this study**
